# Overexpression of sICAM-1 in the Alveolar Epithelial Space Results in an Exaggerated Inflammatory Response and Early Death in Gram Negative Pneumonia

**DOI:** 10.1186/1465-9921-12-12

**Published:** 2011-01-19

**Authors:** Michael P Mendez, Yeni K Monroy, Ming Du, Angela M Preston, Leslie Tolle, Yujing Lin, Kelli L VanDussen, Linda C Samuelson, Theodore J Standiford, Jeffery L Curtis, James M Beck, Paul J Christensen, Robert Paine

**Affiliations:** 1 Division of Pulmonary and Critical Care Medicine, Henry Ford Health System, 2799 West Grand Boulevard, Detroit 48202, USA; 2 Pulmonary Section, Veterans Affairs Health System, 2215 Fulller Road, Ann Arbor 48105, USA; 3 Division of Pulmonary and Critical Care Medicine, University of Michigan, 1500 E. Medical Center Drive, Ann Arbor 48109, USA; 4 Molecular and Integrative Physiology, University of Michigan, 2041 BSRB, 109 Zina Pitcher Place, Ann Arbor 48109, USA; 5 Division of Pulmonary and Critical Care Medicine, University of Utah, Wintrobe Building, Room 701, 26 North 1900 East, Salt Lake City 84132, USA; 6 Pulmonary Section, Veterans Affairs Health System, 500 Foothill Drive, Salt Lake City 84148, USA

## Abstract

**Background:**

A sizeable body of data demonstrates that membrane ICAM-1 (mICAM-1) plays a significant role in host defense in a site-specific fashion. On the pulmonary vascular endothelium, mICAM-1 is necessary for normal leukocyte recruitment during acute inflammation. On alveolar epithelial cells (AECs), we have shown previously that the presence of normal mICAM-1 is essential for optimal alveolar macrophage (AM) function. We have also shown that ICAM-1 is present in the alveolar space as a soluble protein that is likely produced through cleavage of mICAM-1. Soluble intercellular adhesion molecule-1 (sICAM-1) is abundantly present in the alveolar lining fluid of the normal lung and could be generated by proteolytic cleavage of mICAM-1, which is highly expressed on type I AECs. Although a growing body of data suggesting that intravascular sICAM-1 has functional effects, little is known about sICAM-1 in the alveolus. We hypothesized that sICAM-1 in the alveolar space modulates the innate immune response and alters the response to pulmonary infection.

**Methods:**

Using the surfactant protein C (SPC) promoter, we developed a transgenic mouse (SPC-sICAM-1) that constitutively overexpresses sICAM-1 in the distal lung, and compared the responses of wild-type and SPC-sICAM-1 mice following intranasal inoculation with *K. pneumoniae*.

**Results:**

SPC-sICAM-1 mice demonstrated increased mortality and increased systemic dissemination of organisms compared with wild-type mice. We also found that inflammatory responses were significantly increased in SPC-sICAM-1 mice compared with wild-type mice but there were no difference in lung CFU between groups.

**Conclusions:**

We conclude that alveolar sICAM-1 modulates pulmonary inflammation. Manipulating ICAM-1 interactions therapeutically may modulate the host response to Gram negative pulmonary infections.

## Background

Intercellular adhesion molecule-1 (ICAM-1) is an ~100 kDa molecule belonging to the immunoglobulin supergene family. The membrane bound form of this protein (mICAM-1) serves as a counter-receptor for the β2 integrins, CD11a/CD18 (LFA-1) and CD11b/CD18 (Mac-1), found on leukocytes. Interactions with mICAM-1 facilitate leukocyte transmigration across the endothelium [1] and over the surface of alveolar epithelial cells (AECs) in the lung [2]. Studies using gene-targeted mice lacking ICAM-1 or neutralizing antibodies have indicated that ICAM-1 is necessary for normal pulmonary host defense [3-5]. A soluble form of the molecule, soluble intercellular adhesion molecule-1 (sICAM-1), is found in serum and in the alveolar lining fluid [6-8]. sICAM-1 in the alveolar space is likely generated by proteolytic cleavage of mICAM-1 found on type I alveolar epithelial cells [9].

sICAM-1 is normally present in the alveolar lining fluid of both humans and mice [6,7,10-13]. Like mICAM-1, sICAM-1 binds to LFA-1/Mac-1 and not only competes with leukocyte binding to mICAM-1 [14], but also stimulates leukocyte cytokine production [15]. We have previously demonstrated that isolated alveolar epithelial cells (AECs), which express features of the type I cell phenotype, release sICAM-1 in primary culture [7]. However, little is known regarding the physiologic significance of sICAM-1 in the alveolus. Because sICAM-1 is abundant in the alveolar lining fluid, and modulates both leukocyte adhesion and stimulation, sICAM-1 may modulate AEC-leukocyte interactions in the alveolus, and thus play an important role in lung diseases characterized by alveolar inflammation, such as pneumonia and acute lung injury.

Based on these considerations, we hypothesized that overexpression of sICAM-1 in the alveolus would modulate the innate immune response during acute lung inflammation and infection in mice. To address this hypothesis, we designed and characterized a genetically modified mouse that overexpresses sICAM-1 in the alveolus under control of the surfactant protein C promoter (SPC-sICAM-1). We evaluated this mouse using an established model of pulmonary infection with *K. pneumoniae*, comparing survival, cellular accumulation and recruitment, and alveolar macrophage (AM) function in SPC-sICAM-1 and wild-type mice. SPC-sICAM-1 mice demonstrated increased mortality and increased systemic dissemination of organisms compared with wild-type mice, but no change in the burden of organisms within the lung. We also found that SPC-sICAM-1 mice demonstrated exaggerated inflammatory responses compared with wild-type mice. One potential mechanism underlying these differences is sICAM-1's ability to prime alveolar macrophages for elaboration of cytokines in response to LPS.

## Methods

### Animals

Pathogen-free wild-type C57BL/6 mice were obtained from Jackson Laboratories (Bar Harbor, ME) at 6-12 weeks of age. All animals were housed in isolator cages within the Animal Care Facilities at the Ann Arbor Department of Veterans Affairs Research Laboratories. Mice received food and water ad libitum. The experimental protocols were approved by the animal care committees at the University of Michigan and the Veterans Affairs Medical Center.

### Transgenic Mouse Design

**Figure 1:**
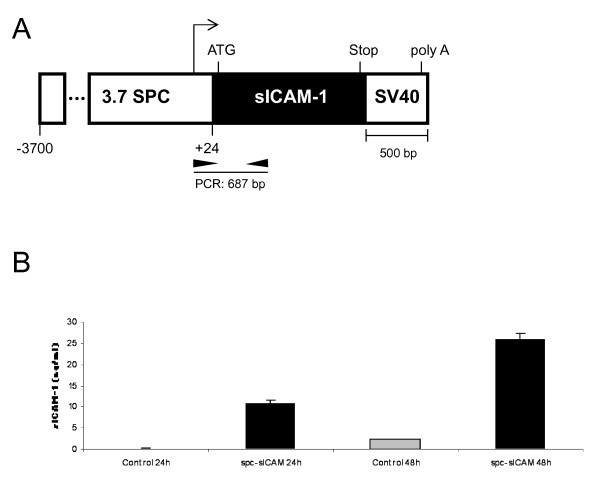
**Design of SPC-sICAM-1 transgene construct and transfection into MLE12 cell line**. The transgene, sICAM-1, was placed under the control of the human SPC promoter (-3700 to +24 bp). The SV40 cassette provided intronic and polyadenylation sequences. The approximate locations of primers for genotyping transgenic mice are indicated (arrowheads) together with the PCR product (a). Function of the transgene was demonstrated by transient transfection of the transgene into MLE12 cells (b). sICAM-1 in the cell culture supernatants was measured at 24 and 48 hrs as shown (n = 3, * P < 0.05).

The backbone of the transgenic construct was pUC18 containing a 3.7 kB human SPC promoter, a multiple cloning site, and SV40 small t-intron and polyadenylation signal, which was kindly provided by Dr. J. Whitsett (Children's Hospital, Cincinnati, OH). The truncated mICAM-1 sequence, with transmembrane and cytoplasmic domains removed, was kindly provided by Dr. D. Wagner (Harvard Medical School, Boston, MA) on a pBluescript backbone [16]. The truncated ICAM-1 cDNA fragment was cut from the pBluescript plasmid with EcoR I and ligated into the multiple cloning site of the pUC18 vector (Figure 1a). The final construct was verified by sequencing. Its functionality was verified by transfection into MLE12 cells (ATCC, Manassas, VA), a cell line derived from human AECs that express SPC, and measurement of sICAM-1 in the cell culture supernatants (Figure 1b). The Nde I/Not I linearized transgene DNA fragment was purified and microinjected into C57BL/6 fertilized eggs as described [17]. Four founders were identified and were mated with wild-type C57BL/6 mice. Of the four founders, two were discarded due to near normal expression of sICAM-1, measured in the bronchoalveolar lavage (BAL). Both of the two remaining founders expressed sICAM-1 in the BAL at high levels but only one transmitted the transgene in the expected fashion (50% transmission rate to offspring). This founder (SPC-sICAM-1) was mated with C57Bl/6 mice to produce F1 generation mice for subsequent experiments.

### Characterization of SPC-sICAM-1 mice

Mice containing the transgene were identified by polymerase chain reaction (PCR). The forward primer was designed specific to the transcription start site of the SPC gene, 5'-CATATAAGACCCTGGTCACACCTGGGAGA-3', and the reverse primer, 5'-TGTGCGGCATGAGAAATTGGCTCCGTGGTC-3', was designed specific to the ICAM-1 cDNA region (product size 687 bp). A PCR primer directed to the endogenous mouse cholecystokinin gene was used as an internal control (forward: 5'-CTGGTTAGAAGAGAGATGAGCTACAAAGGC-3', reverse: 5'-TAGGACTGCCATCACCACGCACAGACATAC-3'; product size 361 bp). The PCR conditions were the same for each primer pair: 92°C for 2 minutes then 94°C for 30 seconds followed by annealing at 65°C for 30 seconds followed by elongation at 72°C for 45 seconds. The latter three steps were repeated for 34 cycles. The reaction was completed at 72°C for 5 minutes. PCR product sizes were analyzed by electrophoresis on a 2.2% FlashGel DNA cassette (Lonza, Rockland, ME) using the FlashGel system (Lonza). Confirmation of lung specific expression was performed by isolating total RNA from lung, spleen, heart, liver, and kidney using the Absolutely RNA Miniprep Kit (Strategene, La Jolla, CA) following the manufacturer's instructions. The purified RNA was subjected to reverse transcriptase PCR with primers specific for the proSPC-sICAM-1 message (forward primer: 5'-ACCTGCAGGTCGACTCTAGAGGATCCC-3'; reverse primer: 5'- TGTGCGGCATGAGAAATTGGCTCCGTGGTC-3'; product size 637 bp; Figure 1a). The real time PCR reaction conditions were as follows: 55°C for 40 minutes, then 95°C for 10 minutes, followed by 95°C for 30 seconds, 60°C for 1 minutes, and 72°C for 30 seconds. The latter three steps were repeated for 34 cycles. The resultant product was then analyzed by electrophoresis on a 2.2% Lonza gel.

### Processing of bronchoalveolar lavage fluid for Western analysis

BAL was performed in transgenic mice and control mice using previously described methods [18]. BAL was performed using five 1-ml aliquots of PBS that were pooled. Typical return was 90-95% of instilled volume. BAL fluid was centrifuged at 500 × *g *for 10 minutes at 4°C to remove whole cells. Diluted proteins from BAL were concentrated using a 100 kD molecular weight cut off centrifugal filter (Millipore). Supernatants were stored at -70°C for subsequent analysis of sICAM-1 by Western Blot.

### Western analysis of sICAM-1

The samples were denatured in sample buffer [2% sodium dodecyl sulfate (SDS), 10% glycerol, 62.5 mM Tris HCl, pH 6.8] at 100°C and separated by SDS-polyacrylamide gel electrophoresis (PAGE) (10% acrylamide) under non-reducing conditions, loading 20 μg of protein in each lane. After PAGE, the separated proteins were electrophoretically-transferred to PVDF membrane (Bio-Rad Laboratories, Richmond, CA). Full range protein molecular weight standards were purchased from Bio-Rad Laboratories. The PVDF membranes were incubated in 5% bovine serum albumin to block nonspecific binding and exposed to rat mAb AB796 (R&D Systems, specific for the extracellular domain of mouse ICAM-1), or control rat IgG_2b _antibody (R&D Systems). The membranes were then incubated with anti-rat secondary antibody conjugated to horseradish peroxidase (Jackson ImmunoResearch Laboratories, West Grove, PA). The membranes were washed extensively in Tris-buffered saline after each step. Subsequently, the blots were developed using a chemiluminescence system (ECL Western Blotting detection system, Amersham, Arlington Heights, IL) according to the manufacturer's recommendations.

### sICAM-1 ELISA

BAL serum and lung homogenates from mice from experimental and control groups were analyzed for total sICAM-1 levels by commercially available ELISA kits (R&D Systems, Minneapolis, MN). The absorbance was measured at 450 nm by a microplate autoreader (BioTek, Winooski, VT), with a correction wavelength set at 570 nm. All measurements were preformed following the manufacturer's instructions, and the final concentrations were calculated by reference to the standard curves.

### *Preparation of *Klebsiella pneumoniae

*K. pneumoniae *strain 43816, serotype 2 was obtained from American Type Culture Collection (ATCC, Manassas, VA). *K. pneumoniae *was grown overnight with aeration in 25 ml of LB broth (Invitrogen, San Diego, CA), at 37°C on a shaker at 300 rpm. The culture was diluted 1:20 and grown for 45 min at 37°C until it reached 0.1 nm OD. Bacteria were then diluted in sterile phosphate-buffered saline (PBS) to the appropriate CFU/ml (2500 or 250 CFU/100 μl) for intranasal inoculation. Bacteria were maintained on ice until inoculation.

### *Inoculation of mice with *K. pneumoniae

Twelve week old transgenic mice and wild-type mice were anesthetized with inhaled isofluorane and inoculated intranasally with 100 μl of the *K. pneumoniae *suspension. Appropriate dilutions of the inocula were plated on LB agar plates to confirm the doses administered. Other groups of mice were not exposed to *K. pneumoniae*, but were inoculated with 100 μl of PBS as negative controls.

### Lung harvest for histological examination

At 24 hours post-inoculation, one mouse from each group was euthanized for lung histology. The lungs were perfused via the right ventricle with DPBS to remove excess blood and inflated with formaldehyde to improve resolution. The lungs and central airways were then removed en bloc and fixed in formaldehyde. After removing the central airways, the lungs were transferred to histocassettes (Fischer Scientific), incubated in formaldehyde, embedded in paraffin and processed for sectioning and staining.

### *Determination of lung CFU and dissemination of *K. pneumoniae

In order to assess the burden of organisms with the lungs, mice were inoculated with *K. pneumoniae *(2500 CFU in100 μl) and euthanized after 24 hours. The pulmonary vascular bed was perfused via the right ventricle with DPBS. Lungs and spleen were removed using sterile technique, and collected in 1 ml and 0.5 ml of 2× Complete Buffer (Roche, Nutley, NJ), respectively. The tissues were then homogenized with an Ultra-Turrax T8 Homogenizer (IKA-Labortechnik, Germany). Aliquots from lungs and spleens were serially diluted in DPBS to 10^-9^. 10 μl of each dilution was plated on LB agar plates (Invitrogen) and incubated at 37°C. Colony counts for each animal were determined after 24 hours. A priori, we defined positive spleen cultures to be > 10 CFU of *K. pneumoniae*.

### *Phagocytosis of *fluorescent beads *by AM*

Mice were inoculated intranasally with 100 μl of 5 × 10^7 ^of FITC-labeled bioparticles (pHrodo Bioparticles Conjugates, Invitrogen). Control mice were inoculated with DPBS. One hour post-inoculation, four mice from each group were euthanized and BAL was obtained as described [19]. Fluorescence intensity of each sample was measured by flow cytometry, using a FACScan cytometer (Becton Dickinson, Mountain View, CA) with CellQuest software. A minimum of 10,000 viable cells was analyzed per sample.

### Differential cell counts in total lung lavage by flow cytometry

Perfused lungs were lavaged with10 ml of Dulbecco's PBS with mM EDTA. Cells were washed with PBS and resuspended at 1 × 10^6 ^cells per ml of PBS. Before addition of antibodies, the samples were prepared with a Live/Dead Fixable Aqua Dead Cell Strain Kit (Invitrogen, San Diego, CA). Fc Block was added to all samples following the manufacturer's instructions to reduce non-specific binding of antibodies to the activated cells. For analysis by flow cytometry, the samples were washed twice in staining buffer (Difco, Detroit, MI), resuspended in staining buffer, and incubated for 30 min at 4°C in the dark with labeled antibodies. The following antibodies were obtained from BD: 1A8 (antimurine Ly-6G; FITC-conjugated) was used to gate on PMN; M1/70 (antimurine CD11b, PerCP-Cy5.5-conjugated) was used to gate on monocytes; and 30-F11 (antimurine CD45; APC-Cy7-conjugated) was used to gate on all leukocytes. The following antibodies were obtained from eBioscience (San Diego, CA): MTS510 (antimurine TLR4; PE-conjugated), N418 (antimurine CD11c; Pacific Blue) was used to gate on mature alveolar macrophages; and HI30 (antimurine CD45; Pacific Blue) was used to gate on all leukocytes. Appropriate isotype-matched controls were used in all experiments. All samples were analyzed on the BD LSR II flow cytometer with 3 lasers (488 nm blue, 405 nm violet, and 633 nm HeNe red). A minimum of 10,000 viable cells was analyzed per sample, first gating on CD45+ cells and second gating on live cells using the Live/Dead Fixable Aqua Dead Cell Strain Kit. Gating on specific leukocytes populations was performed using antibodies described above. Absolute numbers of each subset were determined by multiplying the total cell count by hemacytometer with percentage results from flow cytometry. Data were collected using FACS Diva software with automatic compensation and were analyzed using FlowJo software.

### In vitro stimulation of AM with LPS and recombinant sICAM-1

AM were isolated from wild type C57BL/6 mice by bronchoalveolar lavage with PBS. The AM were plated at a concentration of 100,000 cells/well and allowed to adhere in a 96-well plate for one hour. Cells were incubated individually or in combination with Polymixin B Sulfate (Sigma Aldrich) (50 μg/ml), recombinant sICAM-1 (Stem Cell Technologies) (50 μg/ml), and/or LPS (*Escherichia coli*-derived; Sigma Aldrich) (1 μg/ml). Samples were incubated for 24 hours. All incubations were performed at 37°C and 5% CO2. After the incubation, the media was recovered and the supernatants were analyzed by commercially available ELISA kits (R&D Systems, Minneapolis, MN) for MIP2, KC, and TNF-α. The addition of Polymixin B had no effect on cytokine expression induced by sICAM-1 alone, suggesting that recombinant sICAM-1 was not contaminated with LPS (data not shown).

### Statistical Analysis

Data are expressed as means with standard error of the mean represented by error bars. The data were compared using a two-tailed Student's t-test or a Chi square contingency table. If more than two groups were compared an ANOVA was used. Survival difference between groups was analyzed using Kaplan-Meier curves and Log-rank (Mantel-Cox) Test. Differences were considered statistically significant if *p *values were < 0.05. All statistical analysis was performed with the GraphPad Prism 5 package from GraphPad Software (San Diego, CA).

## Results

### SPC-sICAM-1 transgenic mice overexpress sICAM-1 in the lung

**Figure 2:**
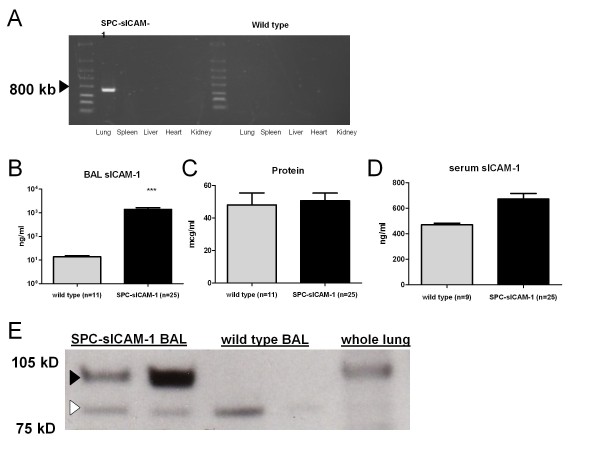
**Characterization of SPC-sICAM-1 transgenic mice**. Transgene-specific primers demonstrated lung-specific expression of the SPC-sICAM-1 transgene in the lungs, with no expression detected in wild-type mice (a). Protein expression in SPC-sICAM-1 in BALF was increased (~2 log fold) over wild type mice (b, *** P < 0.05 by t-test), but did not significantly affect total BALF protein or serum sICAM-1 expression (c, d). Western analysis of BALF demonstrates a larger protein (~100 kDA, black arrowhead) in SPC-sICAM-1 BALF not present in wild type BALF. The lighter bands (white arrowhead) represent endogenous processing of sICAM-1. Whole lung mince from a wild type mouse is included for comparison. Two representative mice are shown for SPC-sICAM-1 (F1 generation) and wild type mice (e).

In order to begin to dissect the contribution sICAM-1 to host defense in the distal lung in the setting of acute infection, we designed a transgenic mouse that would overexpress sICAM-1 in the alveolar space. We chose the human SPC promoter to drive expression of sICAM-1 on a C57BL/6 background using conventional transgenic technology as described in the Materials and Methods section. The founder offspring were grossly indistinguishable from wild-type mice. There was no significant difference in weights or in histologic appearance of the lungs (data not shown). We confirmed lung-specific mRNA expression of the transgene by performing real time RT PCR on the lung and multiple other organs of both SPC-sICAM-1 and wild type mice using a primer set specific to the transgene sequence (Figure 2a). sICAM-1 protein expression in BAL was about 2-log fold higher in SPC-sICAM-1 (1389 ± 237 ng/ml) mice than in wild-type mice (13.7 ± 1.6 ng/ml) (Figure 2b). Increased sICAM-1 protein expression in the lung did not affect either total protein concentrations in BAL or sICAM-1 levels in the serum in transgenic mice compared to wild-type controls (Figure 2c, d).

A western blot of BALF protein from transgenic versus wild-type mice using an anti-ICAM-1 antibody specific to the external domain of mICAM-1 demonstrated a unique100 kDA band (transgene product, black arrowhead Figure 2e) in the transgenic mice, not present in the wild type mice. A slightly lower molecular weight band representing endogenous sICAM-1 was present in both SPC-sICAM-1 transgenic and wild-type mice (white arrowhead Figure 2e). We have previously shown that production of sICAM-1 in the alveolar space of wild-type mice is likely mediated by proteolytic cleavage of mICAM-1 on the surface of type I AEC [9]. In addition to proteolytic-mediated production of sICAM-1, SPC-sICAM-1 mice also generate sICAM-1 through direct release of the transgene protein from type 2 AEC. The transgenic sICAM-1 lacks membrane and cytoplasmic domains and thus is directly released from the cell.

### *SPC-sICAM-1 mice have decreased survival compared to wild-type mice after *K. pneumoniae *infection*

**Figure 3:**
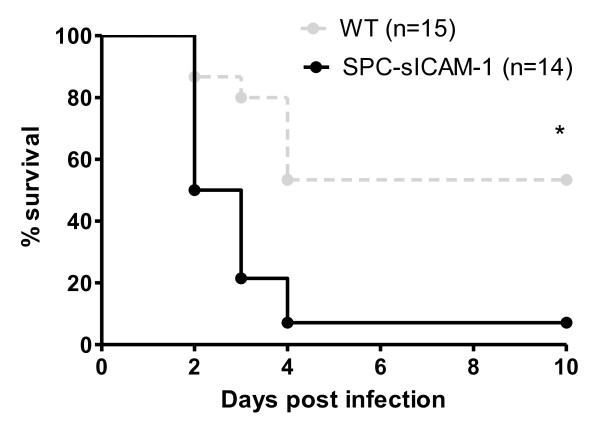
**Overexpression of sICAM-1 in the distal lung results in decreased survival after *K. pneumoniae *infection**. SPC-sICAM-1 mice and wild-type controls were inoculated intranasally with 2500 CFU of *K. pneumoniae *on day 0 and the percentage of mice surviving was determined over time. At 10 days, survival was significantly decreased in the SPC-sICAM-1 mice compared with infected wild-type controls. * P = 0.0012 compared with wild-type control mice.

We have previously shown that mutant mice deficient in mICAM-1 have decreased survival in a model of *K. pneumoniae *pneumonia [20]. Subsequent studies suggested that the loss of ICAM-1-mediated interaction between type I AEC and AM resulted in decreased macrophage phagocytic and bactericidal activities [20]. It is unclear what role sICAM-1 might have in these processes. To explore the effects of sICAM-1 in the distal lung on survival in acute lung infection, we inoculated SPC-sICAM-1 and wild-type mice with 2500 CFU of *K. pneumoniae *and assessed survival over 10 days. sICAM-1 overexpression in the distal lung resulted in greatly decreased survival following intranasal inoculation with *K. pneumoniae *(87% or 6.6-fold decrease) compared to similarly inoculated wild-type mice (Figure 3). Thus, supraphysiologic levels of sICAM-1 in the alveolar space significantly increased mortality in the setting of *K. pneumoniae *infection.

### SPC-sICAM-1 mice infected with K. pneumoniae demonstrate increased systemic dissemination compared to wild-type mice

**Figure 4:**
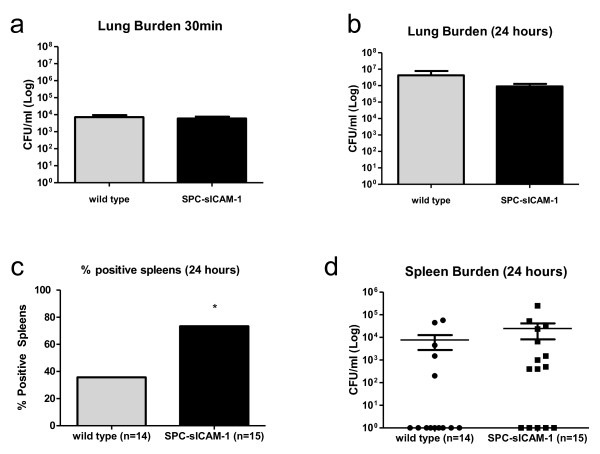
**Increased systemic dissemination, but similar lung burden, occurs 24 hours after *K. pneumoniae *infection**. SPC-sICAM-1 mice and wild-type mice were inoculated intranasally with 2500 CFU of *K. pneumoniae*. After 30 minutes (a) and 24 hours (b), the animals were euthanized, and *K. pneumoniae *CFU were determined in lung homogenates. The percentage of positive spleen cultures and CFU were determined at 24 hours(c, d). Data are expressed as CFU per milliliter (mean ± SEM; n = 3 at 30 minutes; n = 14 for wild type and n = 15 for SPC-sICAM-1 at 24 hours; * P < 0.05 compared with wild-type).

Given the decreased survival of SPC-sICAM-1 mice in a model of *K. pneumoniae *infection, we next assessed the affects of sICAM-1 overexpression on the lung burden and dissemination of bacteria. To confirm consistent, equivalent inoculation, we assessed lung burden 30 minutes after inoculation in some mice and observed no differences in bacterial counts (Figure 4a). After 24 hours, we observed roughly 3-log fold increase in bacterial counts in the lungs of both transgenic and wild-type mice compared to the 1/2 hour time point. Despite the increased mortality in SPC-sICAM-1 mice (Figure 3), there was no difference in the burden of organisms in the lungs or spleens between the groups (Figure 4b, d). However, systemic dissemination, as indicated by positive spleen cultures, was significantly more frequent in the SPC-sICAM-1 mice versus wild-type mice (73% and 36%, respectively), suggesting a defect in the ability of SPC-sICAM-1 mice to contain the infection in the lung (Figure 4c).

### SPC-sICAM-1 mice infected with *K. pneumoniae* have increased cellular recruitment compared to wild-type mice

**Figure 5:**
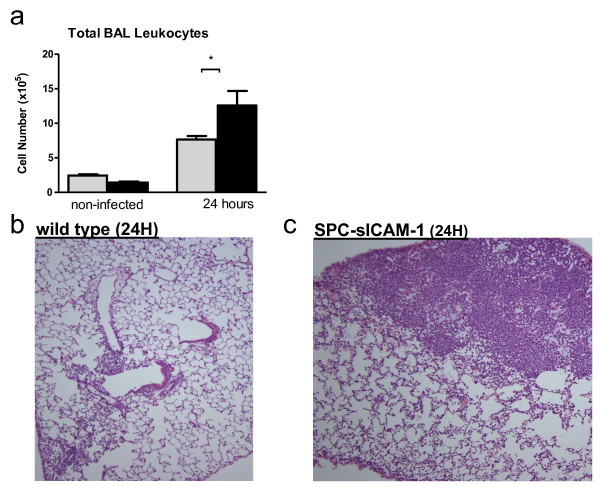
**Increased pulmonary inflammation is observed at 24 hours after *K. pneumoniae *infection in SPC-sICAM-1 mice compared to wild-type mice**. SPC-sICAM-1 mice and wild-type mice were intranasally inoculated with 250 CFU of *K. pneumoniae*. After 24 hours, the animals were euthanized, and whole lung lavage was performed. Whole lung lavage was also collected from mice inoculated with PBS, but not exposed to *K. pneumoniae*, for comparison. Total number of cells was determined by counting with a hemacytometer (A). Representative histologic sections of lungs in wild type (B) and SPC-sICAM-1 mice (C) 24 hours after infection. Data are expressed as mean ± SEM. (n = 6 in all groups; * P < 0.05 compared with wild-type).

**Figure 6:**
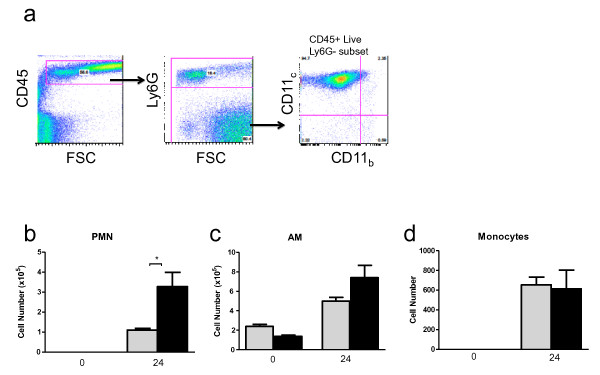
**Increased pulmonary inflammation in SPC-sICAM-1 mice after *K. pneumoniae *infection is due to recruitment of both mononuclear cells and neutrophils**. SPC-sICAM-1 mice and wild-type mice were intranasally inoculated with 250 CFU of *K. pneumoniae*. After 24 hours, the animals were euthanized, and whole lung lavage was performed. Cells were examined by flow cytometry with a gating strategy to identify leukocyte subpopulations as described in materials and methods (a, representative plot, SPC-ICAM-1 at 24 hours). SPC-sICAM-1 had greater accumulation of neutrophils (b), AM (c), and monocytes (d), compared to wild-type at 24 hours, although the differences in AM and monocyte numbers did not reach statistical significance. Data are expressed as mean ± SEM. (n = 6 in all groups; * P < 0.05 compared to wild-type).

We next examined whether leukocyte accumulation in the lung during *K. pneumoniae *infection was affected by sICAM-1 overexpression. After 24 hours, the SPC-sICAM-1 showed a significant increase in BAL leukocytes compared to wild-type mice (Figure 5a). Lung histology showed dense and patchy inflammation in SPC-sICAM-1 mice not seen in wild-type mice (Figure 5b, c) confirming that overexpression of sICAM-1 in the distal lung results in more exuberant inflammatory cell accumulation. To further characterize this inflammation, we examined the recruited cells by flow cytometry using cellular markers specific for AM, monocytes, and neutrophils. SPC-sICAM-1 mice showed significantly increased numbers of neutrophils after 24 hours (Figure 6b, c, d), without significant changes in the numbers of mature AM or monocytes.

To determine a potential mechanism explaining the increased number of acute inflammatory cells in SPC-sICAM-1 mice, we measured chemokines in BAL fluid at 24 hrs. MIP2 and KC were increased in SPC-ICAM-1 mice, although the difference was not statistically significant (54.0 ± 28.8 pg/ml vs 16.5 ± 6.9 pg/ml and 107.8 ± 45.1 pg/ml vs 32.2 ± 7.5 pg/ml, respectively). Thus, high level expression of sICAM-1 in the distal lungs results in increased cellular recruitment in the lung after infection with *K. pneumoniae*.

### SPC-sICAM-1 alveolar macrophage phagocytosis is not impaired compared to wild-type mice

**Figure 7:**
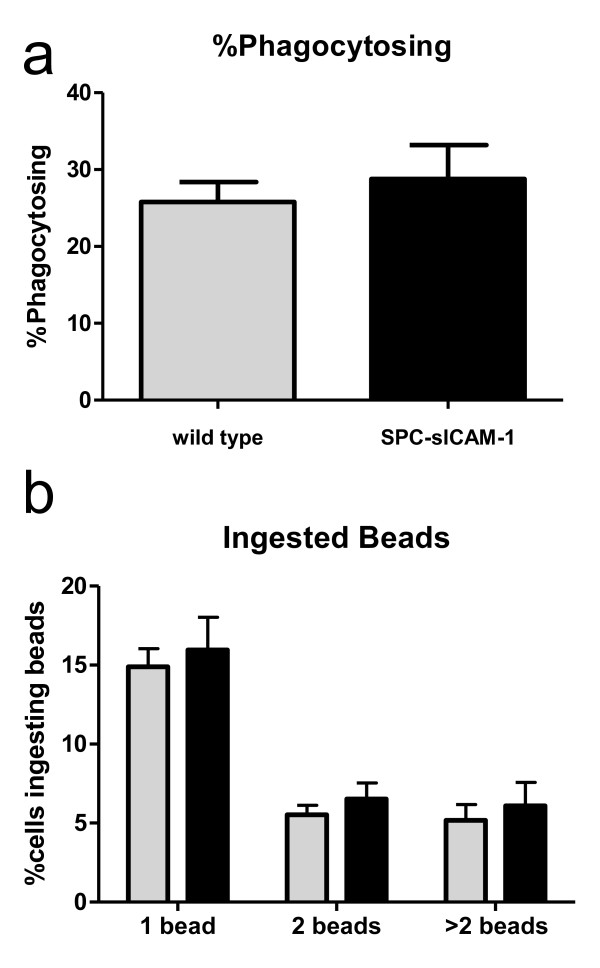
**In vivo phagocytosis of labeled microbeads by AM is similar in SPC-sICAM-1 and wild-type mice**. Mice were lightly anesthetized and intranasally inoculated with 5 × 10^7 ^FITC-conjugated polystyrene microbeads (1.7 micron). After 1 hour, mice were euthanized, and AM were recovered by whole lung lavage. Cells were recovered by centrifugation and examined by flow cytometry. Data are expressed as mean ± SEM for the percentage of AM that have engulfed beads (A) and the percentage of cells ingesting 1, 2, or > 2 beads (B). (n = 5 for all groups; no significant differences between groups)

Having demonstrated a decreased survival and decreased ability to contain bacterial organisms in SPC-sICAM-1 mice, we sought to determine whether AM phagocytosis was compromised in the presence of high levels of sICAM-1. Because our previous studies [20] show the importance of mICAM-1 mediated interaction between AM and AEC in host defense, we assessed AM phagocytosis. SPC-sICAM-1 and wild-type mice were inoculated with fluorescently-labeled polystyrene beads by intranasal instillation. After one hour, AM were collected by lavage and assessed by flow cytometry. Figures 7a and 7b show that the percentage of macrophages phagocytosing one or more beads and the number of beads ingested were similar between SPC-sICAM-1 and wild-type mice. Thus, increased levels of sICAM-1 in the alveolar lining fluid do not modulate macrophage phagocytosis.

### AM incubated with sICAM-1 and LPS in vitro results in synergistic production of TNFα and MIP2

**Figure 8:**
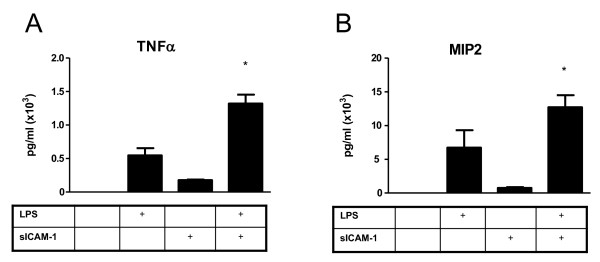
**In vitro stimulation of AM with LPS and recombinant sICAM-1 results in a synergistic increase in TNFα and MIP-2 production from AM**. AM isolated by whole lung lavage from normal wild-type mice were stimulated with LPS (100 μg/ml) and/or recombinant sICAM-1 (50 μg/ml). Both TNFα (A) and MIP-2 (B) were measured by ELISA of cell culture supernatant after a 24 hour incubation. Data are expressed as mean ± SEM. (n = 6 for all groups; * P < 0.05 compared to all other conditions).

Having detected a trend toward increased intra-alveolar cytokine and chemokine levels in SPC-sICAM-1 mice compared to wild-type mice in response to in vivo *K. pneumoniae *infection, we next determined whether sICAM-1 could directly enhance in vitro AM cytokine or chemokine release. AM isolated from wild-type mice were incubated with recombinant murine sICAM-1 and/or LPS. After 24 hours, cell free supernatants were collected and analyzed for TNFα or MIP-2. There was no detectable TNFα or MIP-2 in supernatants from unstimulated AM (Figure 8a, b). As expected, LPS induced expression of both TNFα and MIP-2 above baseline. Recombinant sICAM-1 induced expression of both TNFα and MIP-2, albeit at much lower levels (32% and 11% of LPS induction, respectively). Interestingly, incubation with both LPS and sICAM-1 induced a response from AM that was synergistic. LPS and recombinant sICAM-1 induction of TNFα and MIP-2 was 2.3× and 1.7× greater, respectively, than expected from an additive affect. These data demonstrate that sICAM-1 modulates the AM chemokine and cytokine responses to LPS.

### SPC-sICAM-1 mice infected with *K. pneumoniae* demonstrate a trend toward increased alveolar leak

**Figure 9:**
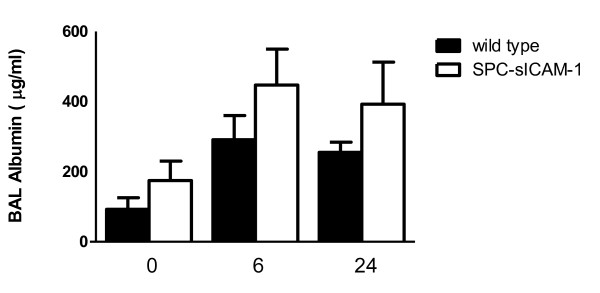
***K. pneumoniae *infection of SPC-sICAM-1 mice may be associated with greater alveolar leak compared to wild type mice**. SPC-sICAM-1 mice and wild-type mice were intranasally inoculated with 250 CFU of *K. pneumoniae*. After 6 and 24 hours, the animals were euthanized, and whole lung lavage was performed. Albumin was measured by ELISA of cell free supernatant. Data are expressed as mean ± SEM. (n = 6 for all groups).

To ascertain whether acute lung injury was associated with increased dissemination and decreased survival in SPC-SICAM-1 mice infected with *K.pneumoniae*, we examined albumin levels in BAL of mice. In these studies, transgenic and wild-type mice were intranasally inoculated with 250 CFU of *K. pneumoniae*. At 6 and 24 hours, BAL was collected and albumin was measured from the cell free supernatant by ELISA. We noted a trend in a sustained increase in albumin levels at 6 and 24 hours in the SPC-sICAM-1 mice compared to the wild type mice (Figure 9). This suggests that alveolar leak may be a plausible mechanism for increased dissemination in the SPC-sICAM-1 mice.

## Discussion

In these studies, we evaluated the effect of lung targeted expression of sICAM-1 in the alveolar space in the context of Gram negative pneumonia. There are several key findings. First, high levels of sICAM-1 in the alveolus increased mortality after *K. pneumonia *infection. Second, this increased mortality was associated with increased systemic dissemination of organisms, without change in the burden of organisms within the lung. Third, high levels of sICAM-1 in the alveolus did not affect AM number, phenotype or phagocytic function. Fourth, high levels of sICAM-1 in the alveolus resulted in enhanced cellular recruitment of acute inflammatory cells to the lung after *K. pneumonia *infection. Finally, sICAM-1 and LPS interact synergistically to increase cytokine elaboration by AMs. Taken together, these findings imply a significant, unique role for sICAM-1 in modulating the inflammatory response to alveolar infections.

In this study, we used transgenic technology to direct expression of the sICAM-1 molecule to the alveolus using the human SPC promoter. The 3.7 kB human SPC promoter has been used successfully to drive expression of GM-CSF in a mouse deficient in GM-CSF to correct the condition of pulmonary alveolar proteinosis in the deficient mice [21]. Others have used the human SPC promoter to direct expression human alpha-1 antitrypsin to the alveolus to assess development of emphysema in a smoking mouse model [22]. We used the same promoter to drive expression of a truncated form of mICAM-1 in the lung. The founder line that was selected for study was morphologically and behaviorally indistinguishable from the wild-type litter mate controls. This founder was specifically chosen due to its high level of sICAM-1 expression found in the BALF compared to wild-type mice (100-fold increase). BALF protein examined by Western Blot demonstrated a discrete band at apparent molecular weight (~100 kD) that was nearly the same as that of mICAM-1 (~105 kDA). The size of endogenous sICAM-1 is ~90 kDA [7,23]. We have previously shown that endogenous sICAM-1 in the alveolus is most likely proteolytically cleaved from mICAM-1 on the surface of type I AEC [9]. ICAM-1 is heavily glycosylated and its apparent molecular weight can vary [24]. Because sequencing confirmed that the transgene actually lacked the intracellular and transmembrane portions of ICAM-1(data not shown), it is most likely that the increased apparent molecular weight of transgenic sICAM-1 is a result of post-translational processing, such as differential glycosylation.

These experiments demonstrate that alveolar sICAM-1 overexpression alters the response to infection. Until now, much of the focus on ICAM-1 in lung inflammation has been related to the membrane-bound form and its role in leukocyte trafficking [4,5,25,26]. mICAM-1 and sICAM-1 are expressed and regulated uniquely by type I AEC [9]. Therefore, we hypothesized that alteration in the amount of sICAM-1 in the alveolus would alter the inflammatory response. Our results demonstrate that excess sICAM-1 in the alveolus in the setting of *K. pneumoniae *infection results in decreased survival. It is possible this effect on mortality might be a result of inhibition of AM-AEC interactions mediated by mICAM-1 due to blockade of AM cell surface ligands by the high levels of sICAM-1. Thus sICAM-1 might be playing a role similar to that of other soluble receptors such as syndecans or receptor for advanced glycation end products (RAGE) [27,28]. However, overexpression of sICAM-1 results in a response that differs in significant ways from that found either in ICAM-1 deficient mice or with antibody-mediated neutralization of ICAM-1 in the lung [4,5]. In contrast to the circumstance in ICAM-1 deficient mice, neither the burden of organisms in the lung nor the ability of AM to phagocytose FITC-labeled beads in vivo was altered by overexpression of sICAM-1. However, despite similar numbers of organisms in the lung, inflammatory cell recruitment was in fact increased in SPC-sICAM-1 mice compared to wild-type mice. One may speculate that subtle impairment of AM activity results in excessive inflammation, which in turn contributes to lung injury, impaired barrier function, and increased systemic dissemination of infection. Our findings highlight the delicate balance required in the lung to both protect from infectious insults and preserve functional barrier to the outside world.

The mechanism(s) of decreased survival in the SPC-sICAM-1 mice in the setting of *K. pneumoniae *are likely complex and related to more than one factor. In our previous work, mICAM-1 deficient mice infected with *K. pneumoniae *also had decreased survival [20]. We demonstrated that bacterial phagocytosis and killing by AM and neutrophils was enhanced by the interaction with mICAM-1 on AEC. We attributed the decreased survival in the ICAM-1 deficient mice to the loss of mICAM-1-mediated interactions between AEC and AM that promote AM lateral migration, phagocytosis and bacterial killing. It is possible that, in mice overexpressing sICAM-1 in the lung, competitive binding of sICAM-1 to the normal counter receptors of mICAM-1 on AM, CD11a/CD18 (LFA-1) and CD11b/CD18 (Mac-1), prevents this important interaction. Interestingly, in the present study we found that supraphysiologic sICAM-1 does not impair AM phagocytosis of fluorescent beads in vivo, suggesting that lateral mobility and phagocytic activity of AM remain largely intact in these mice. This preservation of AM phagocytosis may reflect incomplete blockade of mICAM-1-mediated effects by sICAM-1. Despite relatively normal phagocytosis by AM, the efficiency of bacterial killing in vivo is affected. At 24 hours, there is no significant difference in burden of *K. pneumoniae *between SPC-sICAM-1 and wild type mice, but there is increased accumulation of acute inflammatory cells, including neutrophils, in the lungs of SPC-sICAM-1 mice compared to wild-type mice at 24 hours. Thus the efficiency of bacterial clearance is decreased in the SPC-sICAM-1 mice. We postulate that this enhanced inflammation, coupled with increased TNF-α production by alveolar macrophages, ultimately leads to increased systemic dissemination of infection.

One limitation of our transgenic design is that sICAM-1 is constitutively produced by type II AEC. Thus, the endogenous mechanisms regulating shedding of sICAM-1 form type I AEC become minimized. In this setting, overexpression of sICAM-1 may overwhelm the host's ability to modulate local levels of sICAM-1. This may lead to unchecked activation and subsequently exaggerated inflammation that may be the cause of the decreased survival we observed in the *K. pneumonia *infection.

Previous studies examining the role of ICAM-1 in lung inflammation have focused on the role of ICAM-1 in recruitment of leukocytes and the use of ICAM-1 blocking antibodies or ICAM-1 deficient mice. What has not been addressed is whether there are differential effects of mICAM-1 and sICAM-1 on the inflammatory cascade. In experiments designed to block ICAM-1, it is likely that both mICAM-1 and sICAM-1 being blocked [4,5,25,26]. If sICAM-1 is functionally active, it is important to understand how to contrast its effects on leukocytes with the effects of mICAM-1. In studies using ICAM-1 deficient or mutant mice, the goal has generally been to study a mouse deficient in mICAM-1. However, in some instances, transgenic mice deficient in normal mICAM-1 are still capable of expressing sICAM-1, possibly through alternative splicing and subsequent cleavage [29]. In order to separate the roles of mICAM-1 and or sICAM-1 in the alveolar or vascular compartments, it may be necessary to reintroduce either sICAM-1 or a 'noncleavable' membranous ICAM-1 into ICAM-1 knockout mice.

## Conclusions

In summary, our study shows that overexpression of sICAM-1 in the alveolus has a major impact upon host defense in the setting of *K. pneumoniae *infection. In combination with previous data, our data demonstrate that sICAM-1 has functional effects that influence cellular recruitment and AM activation in the setting of acute infection. This suggests that an appropriate balance of ICAM-1 with an optimal amount of local sICAM-1 in the alveolus may be critical for optimal AM function, activation, and leukocyte recruitment.

## Competing interests

The authors declare that they have no competing interests.

## Authors' contributions

MPM conceived and designed the study, acquired and analyzed data, and prepared manuscript. YKM characterized transgenic phenotype, performed survival, ELISA, phagocytosis, in vitro assays, Western analysis experiments, and acquired and analyzed data. MD assisted with characterization of transgenic phenotype. AP performed survival experiments and assisted with acquisition and interpretation of data. LBT and YL assisted with maintenance of mouse colony and performed survival experiments. KLV and LCS assisted with design and creation of transgenic mouse and analysis of transgenic phenotype. TJS assisted with design and analysis of in vitro assays, dissemination experiments, and analysis of data. JLC designed flow cytometric assays to characterize recruitment of inflammatory cell population and analysis of data. JMB assisted with design of study, analysis of data, and manuscript preparation. PJC assisted with design of all experiments, analysis of data, and manuscript preparation. RP assisted with conception of design of study, analysis of data, and manuscript preparation. All authors approved the final manuscript.
